# Thermostable endoglucanases in the liquefaction of hydrothermally pretreated wheat straw

**DOI:** 10.1186/1754-6834-4-2

**Published:** 2011-01-26

**Authors:** Nóra Szijártó, Emma Horan, Junhua Zhang, Terhi Puranen, Matti Siika-aho, Liisa Viikari

**Affiliations:** 1 University of Helsinki, Department of Food and Environmental Sciences, P.O. Box 27, FIN-00014, Helsinki, Finland; 2 Roal Oy, Tykkimäentie 15, FIN-05200, Rajamäki, Finland; 3 VTT Technical Research Centre of Finland, PO Box 1000, FIN-02044, Espoo, Finland

## Abstract

**Background:**

Thermostable enzymes have several benefits in lignocellulose processing. In particular, they potentially allow the use of increased substrate concentrations (because the substrate viscosity decreases as the temperature increases), resulting in improved product yields and reduced capital and processing costs. A short pre-hydrolysis step at an elevated temperature using thermostable enzymes aimed at rapid liquefaction of the feedstock is seen as an attractive way to overcome the technical problems (such as poor mixing and mass transfer properties) connected with high initial solid loadings in the lignocellulose to ethanol process.

**Results:**

The capability of novel thermostable enzymes to reduce the viscosity of high-solid biomass suspensions using a real-time viscometric measurement method was investigated. Heterologously expressed enzymes from various thermophilic organisms were compared for their ability to liquefy the lignocellulosic substrate, hydrothermally pretreated wheat straw. Once the best enzymes were identified, the optimal temperatures for these enzymes to decrease substrate viscosity were compared. The combined hydrolytic properties of the thermostable preparations were tested in hydrolysis experiments. The studied mixtures were primarily designed to have good liquefaction potential, and therefore contained an enhanced proportion of the key liquefying enzyme, EGII/Cel5A.

**Conclusions:**

Endoglucanases were shown to have a superior ability to rapidly reduce the viscosity of the 15% (w/w; dry matter) hydrothermally pretreated wheat straw. Based on temperature profiling studies, *Thermoascus aurantiacus *EGII/Cel5A was the most promising enzyme for biomass liquefaction. Even though they were not optimized for saccharification, many of the thermostable enzyme mixtures had superior hydrolytic properties compared with the commercial reference enzymes at 55°C.

## Background

Novel transportation fuels produced from renewable resources hold increasing promise as environmentally friendly alternatives to petroleum-derived vehicle fuels. Advanced biofuels from non-food plant materials, such as dedicated crops or byproducts of existing agricultural production, are of particular interest, as they represent an ecologically and ethically more acceptable solution [1]. Technologies for the conversion of lignocellulosic substrates to ethanol are being tested in pilot (for example, by SEKAB in Sweden and BioGasol/DTU in Denmark) and demonstration (for example, Abengoa in Spain and Inbicon in Denmark) scales [2]. Nevertheless, the production of lignocellulosic ethanol still needs technological improvements to become economically viable.

Intensive research carried out during the past two decades in the field of lignocellulose pretreatment has lead to the successful development of efficient physicochemical technologies that can open up the complex structure of lignocellulosic substrates, and thus make cellulose embedded in the matrix of hemicelluloses and lignin more accessible to biological conversion [3,4]. Dedicated enzymes are also available on the market to convert both cellulose and hemicellulose to monomeric sugars [5-7], which can be readily taken up and converted to ethanol by, for example, yeasts in anaerobic fermentations.

One of the single most important process parameters that affect the efficiency of the conversion process is the solid content used. Low substrate loadings are connected with large processing volumes, and result in low product concentrations in the broth, leading to increased capital (large equipment size) and energy costs (mixing, heat transition, product recovery). Until recently, dry matter (DM) content percentage of <10% have been routinely used. When aiming for a final ethanol concentration in the fermentation broth of >4% w/w, which is considered as a prerequisite for feasible large-scale distillation process, the dry-matter content has to exceed 15% for most lignocellulosic substrates [8,9]. High-solid loadings in the enzymatic hydrolysis have been used in many recent studies, including 30% steam pretreated corn stover [10], 30% hot water-treated or steam-exploded olive tree biomass [11], or 40% hydrothermally pretreated wheat straw [12]. For most technical substrates, these high-solid contents create an environment in which practically no free water exists in the pretreated material, and the slurry becomes difficult to handle. The poor mass-transfer conditions can be improved by means of a partial enzymatic hydrolysis stage (liquefaction), where the structured, porous and water-absorbing high-solid substrate is converted to a more flowable fluid. Recent advances to replace the conventional stirring systems by gravimetric mixing have also helped to overcome this problem and made the liquefaction of pretreated lignocellulose possible at up to 40% initial DM content [8,12].

Implementation of the liquefaction stage at elevated temperatures, when the material properties and reaction kinetics are more favourable, is expected to bring further improvements to the processes. At higher temperatures, viscosities are inherently lower and reaction rates are higher, potentially reducing processing times and leading to lower investment and energy costs. Integration of a high-temperature pre-hydrolysis step in the process sequence aimed at rapid liquefaction of the high-solid slurry is considered an attractive way to overcome the problems associated with high initial solids contents [13]. Several potential thermostable enzymes acting on lignocellulosic substrates at elevated temperatures have recently been characterized [13,14].

Rheological studies are difficult to perform in lignocellulose suspensions because of the non-homogenous nature of the substrate, comprising a wide range of particle sizes and shapes, with various interactions occurring between the particles and the surrounding fluid. However, a variety of rheological instruments and tools (for example, vanes, parallel plates, torque rheometer) have been used to study the rheology of concentrated lignocelluloses using mainly pretreated corn stover as the substrate (5% to 30% w/w DM). The few fundamental studies carried out to date were consistent in observing a strongly shear-thinning, viscoelastic behaviour, with a concentration-dependent yield stress, which could be adequately described by various rheological models, including the Bingham, Casson or the Herschel-Buckley models [15-19]. The phenomena of decreasing viscosity during lignocellulose hydrolysis has recently been studied [20,21], and it has been concluded that the dominant rheological effect of saccharification is caused by material dilution as mass is transferred from a solid to a liquid phase. However, the traditional approach used in these studies (samples collected at specific time points during hydrolysis experiments and subjected to rheological measurements in a separate instrument) is not convenient for observation and/or investigation of rapid changes in the rheological behaviour of the substrate. Recently, a rotational viscometer (Rapid Visco™ Analyzer, RVA) allowing real-time measurement of substrate viscosity was used to follow the initial phase of enzymatic hydrolysis [22]. In this method, the hydrolysis reaction was performed in the viscometer under fully controlled temperatures and agitation rates, and the viscosity of the substrate was monitored continuously.

In the present study, we compared novel thermostable enzymes (three cellobiohydrolases (CBHs), two endoglucanases (EGs), two xylanases and a β-glucosidase (βG)), primarily for their ability to reduce the viscosity of pretreated lignocellulose at high initial solids. Wheat straw, which is an abundant and promising raw material for second-generation ethanol production, was used as the lignocellulose substrate. Viscosity measurements (RVA method) focused on the early stage of the hydrolysis, where changes in the rheological behaviour of the substrate are most pronounced. Tailored mixtures containing an enhanced ratio of the best-performing liquefying enzyme were prepared and evaluated for their hydrolytic performance at different temperatures.

## Methods

### Substrate

Hydrothermal treatment of wheat straw (*Triticum aestivum *L.) was carried out under previously optimized conditions [8] at Inbicon A/S (Fredericia, Denmark). After pre-soaking in 3 g/l acetic acid at 80°C for 10 minutes the feedstock was steamed at 195°C for 12 minutes in a continuous reactor operated with a feed rate of 50 kg/hour and a straw:water ratio of 1:5. The separated solids were washed, pressed, and stored frozen at -20°C in aliquots until use. The DM content of the material was 34.0% as determined after overnight drying at 105°C. The carbohydrate composition was analyzed after total acid hydrolysis [23,24]. Monosaccharide sugars from the acid hydrolysate were determined by high-performance liquid chromatography (Dionex, Sparta, NJ, USA) [24]. The glucan content of the solids was 58.9% of the DM, comprising 94% of all carbohydrates present. The major hemicellulose sugar was xylose (3.6% of DM).

### Enzymes

The thermostable glycosyl hydrolases were kindly provided by Roal Oy (Rajamäki, Finland): three CBHs originating from strains of *Acremonium thermophilum, Chaetomium thermophilum and Thermoascus aurantiacus*; two EGs originating from strains of *T. aurantiacus *and *A. thermophilum*; two xylanases originating from strains of *T. aurantiacus *and *Nonomuraea flexuosa*; and a βG from *A. thermophilum*. The enzymes (Table 1) were heterologously produced in a genetically modified *Trichoderma reesei *strain, deficient in the four major native cellulases (*Δcbh1*/*cel7A*, *Δcbh2*/*cel6A*, *Δegl1*/*cel7B*, *Δegl2*/*cel5A*), under the control of the strong *cbh1/cel7A *promoter of the host fungus [25]. The Ta CBHI/Cel7A construct contained the cellulose-binding module (CBM) from *T. reesei *CBHI/Cel7A, and Ta EGII/Cel5A contained the CBM from *C. thermophilum *Ct CBHI/Cel7A. The crude culture supernatants were used without further purification in the liquefaction and saccharification studies. The purity of the preparations was checked by SDS-PAGE analysis using a ready-made gel with 12% Tris-HCl (Bio-Rad, Perth, UK) and Coomassie staining, according to the manufacturer's instructions. The CBHI enzymes have been described in detail by Voutilainen *et al. *[14], and the use of most of the enzymes in thermophilic mixtures discussed by Viikari *et al. *[13].

**Table 1:** The thermostable enzymes used.

Enzyme (traditional name/family based nomenclature)	Source (organism)	Short name (abbreviation used in the text)
CBHI/Cel7A	*Acremonium thermophilum*	At CBHI/Cel7A
CBHI/Cel7A	*Chaetomium thermophilum*	Ct CBHI/Cel7A
CBHI/Cel7A^1^	*Thermoascus aurantiacus*	Ta CBHI/Cel7A
EGII/Cel5A^2^	*T. aurantiacus*	Ta EGII/Cel5A
EGV/Cel45A	*A. thermophilum*	At EGV/Cel45A
XYL/Xyn10A	*T. aurantiacus*	Ta XYL/Xyn10A
XYL/Xyn11A	*Nonomuraea flexuosa*	Nf XYL/Xyn11A
βG/Cel3A	*A. thermophilum*	At βG/Cel3A

^1^Expressed with CBM from *Trichoderma reesei *CBHI/Cel7A

^2^Expressed with CBM from *Chaetomium thermophilum *CBHI/Cel7A

Commercial enzymes (Celluclast 1.5L and Novozym 188l Novozymes A/S, Bagsværd, Denmark) were used as the reference cellulase and supplementary β-glucosidase preparation, respectively.

Enzyme activities were determined at pH 5.0 and 50°C. The filter paper activity (FPA) was determined by the release of reducing sugars from Whatman number 1 filter paper using 2,4-dinitrosalicylic acid (DNS) according to the standardized IUPAC FPA procedure [26]. The EG activity was assayed as the release of reducing sugars from hydroxyethyl cellulose (HEC) using DNS according to the IUPAC procedure [26]. The CBH activity was measured on 1 mmol/l 4-methylumbelliferyl-β-lactoside (MUL) using 100 mmol/l glucose as a β-glucosidase inhibitor, and subtracting the activity of other enzymes (detected in the presence of 5 mmol/l cellobiose) from the value obtained without cellobiose inhibition [27]. Xylanase activity was determined according to Bailey *et al. *using birchwood xylan as substrate [28]. The β-glucosidase activity was assayed by measuring the release of *p*-nitrophenol from *p*-nitrophenyl-β-d-glucopyranoside (PNPG) [29]. Activities were reported as nanokatal (nkat) per mg, except for the FPA, which was reported as filter paper units (FPU) per mg of enzyme.

The protein contents were determined (DC Protein Assay, Bio-Rad Laboratories, Hercules, CA, USA) after precipitation of the proteins from the sample by acetone and using bovine serum albumin as a standard.

### The viscometric (RVA) method

Liquefaction studies were carried out in a microprocessor-controlled rotational viscometer (Rapid Visco Analyzer, model RVA-4l Newport Scientific Pvt. Ltd., Warriewood, Australia) operated by a personal computer running Thermocline for Windows software (version 2.2; Newport Scientific Pvt. Ltd., Warriewood, Australia) as described previously [22].

Enzymatic liquefaction trials were carried out in 50 g of 15% w/w DM wheat-straw suspension prepared in 0.05 mol/sodium citrate buffer pH 5.0. The test temperature was, in general, 50°C, but in temperature-profiling experiments, higher temperatures (up to 75°C) were also tested. Because of the high-solid content, the suspension was prepared on a per-weight basis, and measured directly into the disposable aluminium container of the instrument. The container was then placed in the temperature-controlled copper block of the RVA device, and after the set temperature had been reached, the reaction was started by the addition of 3 mg enzyme per gram dry substrate. In all cases, the added enzyme was made up with buffer to bring the reaction mixture to the target initial solids content (15%) and reaction load (50 g). The suspension was mixed throughout the test by a plastic paddle immersed from the top at a constant rate (100 rpm) and the viscosity of the sample was detected through continuous monitoring of the torque required to maintain the paddle speed at the set value. Apparent viscosities (cP; 1 cP = 1 mPa·s) were read at intervals of 4 seconds for 1 hour, and recorded by the computer.

### Hydrolysis experiments

To study the combined hydrolytic efficiency of thermostable enzymes with enhanced liquefaction ability, hydrolysis experiments were carried out with tailored mixtures containing a relatively high proportion of the best-performing liquefying enzyme. To avoid limitations due to inadequate mixing, the hydrolysis experiment was carried out at a reduced substrate loading using 2% w/w solids (DM) in 10-ml plastic tubes (Falcon; BD Biosciences, San Jose, CA, USA) with 5 ml working volumes. The substrate was distributed into the tubes as a thoroughly mixed suspension prepared in 0.05 mol/l sodium citrate buffer pH 5.0. To avoid loss of sugars due to possible microbial contamination, the reaction mixture also contained 0.02% sodium azide. The suspension was prepared to give the target initial solid content and reaction volume in buffer after the enzyme addition. The enzyme dosage was based on the protein content, and fixed at 8 mg (or 7 mg in controls) per gram DM of substrate. Appropriate reference samples were prepared and run together with the experimental series.

Reaction mixtures were incubated at controlled temperatures (35°, 45, 55 and 60°C) in a shaking incubator (200 rpm) with the tubes positioned diagonally to ensure efficient mixing. Duplicate tubes were prepared for each test point (1, 3, 6, and 24 hours) and the whole reaction mixture was taken as the sample. The enzymatic hydrolysis was stopped by boiling the tubes for 10 minutes. After cooling to room temperature, the clear supernatants of samples were collected and analysed for reducing sugars by the dinitrosalicylic acid method of Miller [30], using d-glucose as a standard. Selected samples were analyzed by gas chromatography (GC) with a flame ionization detector (model 6890N with HP-5 column; Agilent Technologies, Santa Clara, CA, USA) as described by Blakeney *et al. *[31] before and after mild acid hydrolysis according to Puls *et al. *[23].

## Results

### Activities of the thermostable enzymes

**Figure 1:**
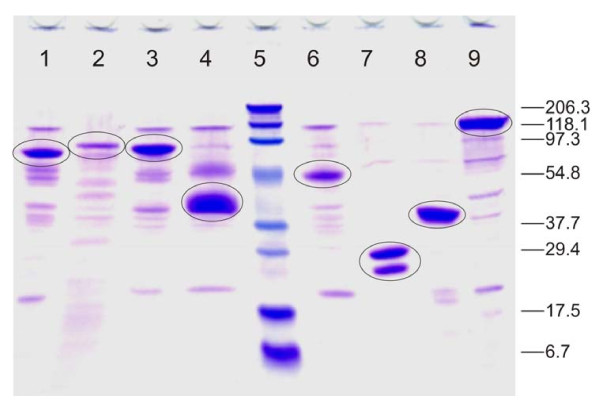
**SDS-PAGE analysis of the thermostable preparations**. Lane 1 = Ta CBHI/Cel7A (6 μg); lane 2 = Ct CBHI/Cel7A (8 μg); lane 3 = At CBHI/Cel7A (5 μg); lane 4 = At EGV/Cel45A (4 μg); lane 5 = protein standards (Bio-Rad 161-0318); lane 6 = Ta EGII/Cel5A (5 μg); lane 7 = Nf XYL/Xyn11A (5 μg); lane 8 = Ta XYL/Xyn10A (5 μg); lane 9 = At βG/Cel3A (4 μg). The Bio-Rad ready-made gel was run at 200V and 100 mA for 1.5 hours and stained with Coomassie for 1 hour. The main protein bands in each preparation representing the heterologously expressed thermostable enzymes are marked. In lane 7, the double band represents the two forms of Nf XYL/Xyn11A: with and without the linker.

Thermostable glycosyl hydrolases from different parent organisms, representing various families of cellulases and xylanases and expressed in *T. reesei*, were chosen for liquefaction and hydrolysis tests (Table 1). The activity profiles of the *T. reesei *culture filtrates containing the heterologously expressed thermostable enzymes (Table 2) were in good agreement with the SDS-PAGE analysis (Figure 1), showing that the target proteins were the main components in the preparations. The double band of the xylanase preparation represents the two forms of NfXYL/Xyn11A, with and without the linker [32]. CBH activity was high in Cel7A preparations, EG activity was high in Cel45A and especially in Cel5A, and β-glucosidase activity was most pronounced in Cel3A. The xylanase background activity varied, but was significant in most of the preparations, because of the high expression level of native *T. reesei *xylanase genes. As expected, none of the enzymes was able to give a high FPA when used individually; the FPA assay is a short hydrolysis test using an insoluble cellulose substrate and therefore, the synergistic action of various glycosyl hydrolases acting in concert is essential to obtain efficient solubilization under the assay conditions.

**Table 2:** The activities of the enzyme preparations.

Enzyme	Specific activities against different substrates (FPU/mg, nkat/mg)				
	
	FPA	HEC	MUL	BX	PNPG
At CBHI/Cel7A	0.17	10.4	12.6	671	4.2
Ct CBHI/Cel7A	BDL^1^	2.0	22.9	72.2	0.1
Ta CBHI/Cel7A	0.05	7.3	10.3	722	2.3
Ta EGII/Cel5A	0.19	1479	BDL^1^	6385	20.7
At EGV/Cel45A	BDL^1^	95.1	BDL^1^	455	1.2
Ta XYL/Xyn10A	BDL^1^	6.0	BDL^1^	7433	2.6
Nf XYL/Xyn11A	BDL^1^	2.5	BDL^1^	5651	1.3
At βG/Cel3A	BDL^1^	27.3	BDL^1^	1602	2348
Celluclast	0.37	129.1	ND^2^	ND^2^	ND^2^
Novozym	ND^2^	5.2	ND^2^	ND^2^	80.5

^1^Below detection limit

^2^Not determined

The hydrolytic activities were measured against filter paper activity (FPA; measuring overall cellulase activity), hydroxyethyl cellulose (HEC; endoglucanase activity), MUL (cellobiohydrolase activity), Birchwood xylan (BX; xylanase activity) and *p*-nitrophenyl-β- d-glucopyranoside (PNPG; β-glucosidase activity).

### Liquefaction of lignocellulose by the thermostable enzymes

**Figure 2:**
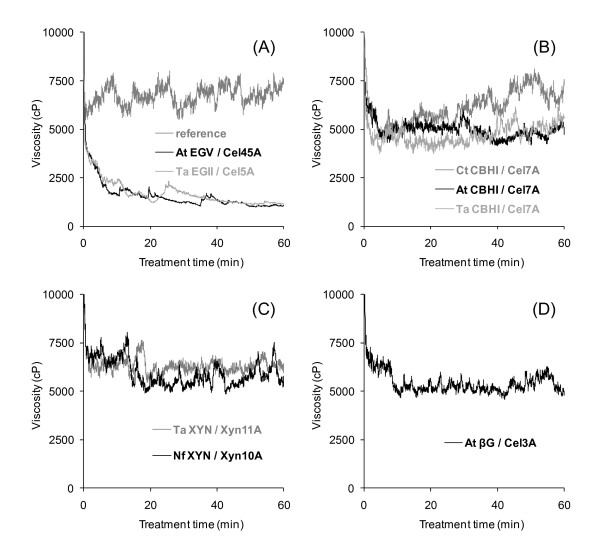
**Comparison of thermostable enzymes in the liquefaction of high-solids lignocellulose**. The effect of (A) endoglucanases Ta EGII/Cel5A and At EGV/Cel45A; (B) cellobiohydrolases At CBHI/Cel7A, Ct CBHI/Cel7A and Ta CBHI/Cel7A; (C) xylanases Ta XYL/Xyn10A and Nf XYL/Xyn11A; and (D) β-glucosidase At βG/Cel3A (3 mg enzyme of each per gram substrate dry matter) was studied with a viscosity of 15% w/w of hydrothermally pretreated wheat straw prepared in 0.05 mol/l sodium citrate buffer (pH 5.0) in a viscosity analyser (100 rpm, 50°C). For the reference solutions, the suspension was made up to the same solids content and incubated with mixing without enzyme addition.

The previously applied viscometric technique (Rapid Visco Analyzer) to monitor enzymatic liquefaction of high-solid lignocelluloses was used to evaluate and compare the ability of selected thermostable enzymes to reduce the viscosity; i.e. increase the fluidity of the pretreated wheat straw substrate (Figure 2). Because of their different activity patterns, the enzymes dose was based on the protein content. The viscosity trials were carried out to compare the behaviour of various enzymes in short-time hydrolytic reactions and not to give absolute viscosity values.

Of the studied enzymes, the EGs were clearly the most efficient in rapidly reducing the viscosity of the biomass suspension at 50°C (Figure 2A), whereas the CBHs (Figure 2B), xylanases (Figure 2C) and β-glucosidase (Figure 2D) had no pronounced effect on the viscosity of the substrate. Differences between the representatives of the two EG families at 50°C were insignificant. The random disruptions observed in the viscosity curves (probably caused by larger aggregated particles in the suspension) did not affect the evaluation of the results; the different liquefaction performance represented by the various types of enzymes was evident. Parallel experiments confirmed the reproducibility of the measurements.

**Figure 3:**
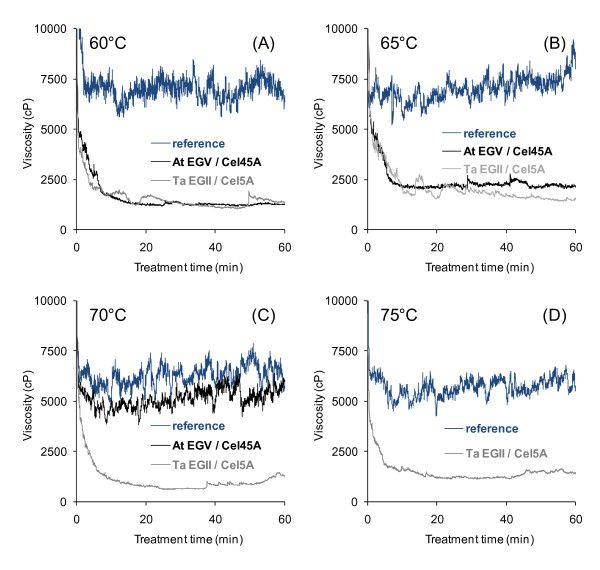
**Temperature profiles of thermostable endoglucanases**. The reduction of substrate viscosity by endoglucanases Ta EGII/Cel5A and At EGV/Cel45A was studied at (A) 60°C, (B) 65°C, (C) 70°C, (D) 75°C in a viscosity analyser. Reaction conditions other than the temperature were as in Figure 2.

Temperature profiles of the thermostable EGs revealed that the *T. aurantiacus *EGII/Cel5A was clearly more thermotolerant than the *A. thermophilum *EGV/Cel45A (Figure 3), with decrease in viscosity still evident at 75°C. Thus, Ta EGII/Cel5A was chosen as the enzyme with the highest potential for liquefaction at high temperatures.

### Hydrolytic performance of the thermostable enzymes in mixtures

**Figure 4:**
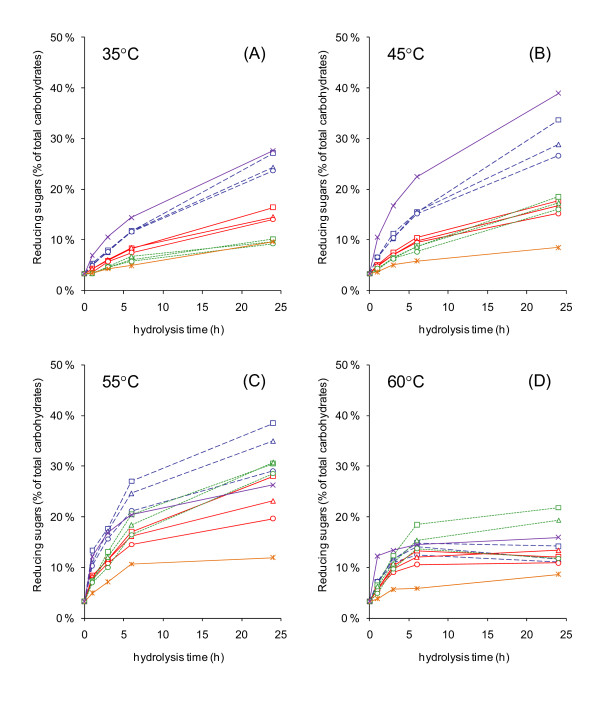
**Hydrolytic performance of the different CBH and XYL enzymes in mixtures**. The substrate, 2% (w/w) hydrothermally pretreated wheat straw, was prepared in 0.05 mol/l sodium-citrate buffer (pH 5.0) and hydrolysed at (A) 35°C, (B) 45°C, (C) 55°C and (D) 60°C. Experiments contained (per gram substrate dry matter (DM)) 3 mg of Ta EGII/Cel5A; 3 mg of the various CBHs: At CBHI/Cel7A (---), Ct CBHI/Cel7A (--) or Ta CBHI/Cel7A (···); and 1 mg xylanase: Ta XYL/Xyn10A (□) or Nf XYL/Xyn11A (Δ). Xylanase-free controls (○) were run in all series. All tubes were supplemented with 1 mg At βG/Cel3A per gram substrate DM. The thermostable reference contained (per gram substrate DM) 7 mg Ta EGII/Cel5A and 1 mg At βG/Cel3A (--*--). Commercial reference was prepared using (per gram substrate DM) 7 mg of Celluclast with 1 mg of Novozym (--x--). The results are based on mean values of DNS analysis of duplicate tubes. The standard deviation of the measurements was consistently <5%.

Ideally, the liquefying enzyme should also contribute to the saccharification of the substrate, and thus reduce the overall enzyme requirement. Because of the synergistic behaviour of cellulases, it was expected that the use of a tailored mixture already present in the pre-hydrolysis step would enhance the hydrolytic potential of the liquefying enzyme. Thus, the combined hydrolytic performance of the thermostable enzymes was studied in mixtures containing an enhanced proportion of the key liquefying enzyme, Ta EGII/Cel5A, in various ratios with the other components. Based on protein content, the mixtures contained the same amount of EG as used in the RVA studies (3 mg per gram dry substrate) to ensure a liquefaction that was at least as efficient as previously obtained. The EG:CBH ratio was kept constant (1:1), and the mixtures were supplemented with the xylanase and β-glucosidase preparations (Figure 4). The mixtures were tested at temperatures relevant for the various process stages: 55°C (or 60°C) for the liquefaction, 45°C for the saccharification, and 35°C for the simultaneous saccharification and fermentation.

The hydrolysis experiments were carried out with a reduced solids content (2% (w/w DM), which allowed uniform distribution of the material as a suspension to tubes using a pipette, and ensured appropriate mass transfer conditions in the experimental tubes during the tests. The relative hydrolytic efficiencies between the studied mixtures were not expected to alter at different consistencies, and therefore the mixture found to perform the best under low-solid conditions was also expected to outperform the other mixtures when the content of solids was increased.

A steady increase in the hydrolytic efficiency of the various mixtures was observed as the temperature increased from 35°C to 55°C. Within this temperature range, enzyme mixtures containing At CBHI/Cel7A resulted in the highest degree of hydrolysis. Mixtures containing Ct CBHI/Cel7A were clearly superior to those containing Ta CBHI/Cel7A at 35°C, but as the temperature increased, the latter gradually took over. At 45°C, the enzyme cocktails prepared using Ta CBHI/Cel7A or Ct CBHI/Cel7A were fairly similar, whereas at 60°C, the mixtures containing Ta CBHI/Cel7A were superior, even to those containing At CBHI/Cel7A. However, the saccharification levels obtained at 60°C were far below those obtained at 55°C. At 55°C, several of the studied combinations of thermostable enzymes were superior to the commercial reference mixture Celluclast supplemented with Novozym at an appropriate ratio.

Regardless of the sources of the CBH, all mixtures performed better when they were supplemented by xylanase, with mixtures containing Ta XYL/Xyn10A being consistently superior to those containing Nf XYL/Xyn11A. In addition to enhanced release of xylose, the presence of xylanase also resulted in improved solubilization of cellulose (Table 3). A considerable amount of the released sugars was present as oligomers.

**Table 3:** Product profiles obtained after enzymatic hydrolysis using enzyme mixtures.

Composition of enzyme mixture, mg per gram solids DM					Monomeric sugars, % of theoretical maximum			
					Glucose		Xylose	
Ta EGII	At CBHI	At βG	Nf XYL	Ta XYL	1	2	1	2
3 mg	3 mg	1 mg	-	-	23.5	31.2	20.7	31.6
3 mg	3 mg	1 mg	1 mg	-	25.0	33.9	26.8	40.4
3 mg	3 mg	1 mg	-	1 mg	27.8	39.2	30.5	50.9
7 mg	-	1 mg	-	-	8.3	11.3	13.4	19.3

Carbohydrate analysis by GC of samples collected after 24 hours of hydrolysis at 55°C of 2% w/w hydrothermally pretreated wheat straw in 0.05 mol/l sodium citrate, pH 5.0, using different combinations of thermostable enzymes. Samples were analysed before (1) and after (2) mild acid hydrolysis.

## Discussion

The viscosity of lignocellulosic substrates is known to decrease as a result of cellulolytic activity, with the most probable reason being the collapse of structure and subsequent loss of water-binding capacity upon cellulose degradation [21]. The phenomenon is well documented in the literature, but information on the individual role of the various types of lignocellulose-degrading enzymes in the liquefaction remains limited. Our viscosity trials were carried out to compare the enzymes in short-time hydrolytic reactions.

The RVA viscometer is an approved apparatus for testing the viscous properties of starch, grain and flour, and formulated, extruded or cooked food. Recently, it has also been used for malted grains to mimic the liquefaction process and to identify the mashing properties of grains [33,34]. Based on these applications, the RVA viscometer was considered to suit to other heterogeneous materials containing soluble and insoluble compounds, such as pretreated lignocellulose, at high consistencies. The studies were aimed to compare the effects of individual enzymes in the improvement of fluidity (reduction of viscosity) of the high-solid substrate at a phenomenal level, and were not meant to discuss the changes in the material properties in terms of absolute viscosity values.

Of the studied enzymes, the EGs were clearly the most efficient in rapidly reducing the viscosity of the biomass suspension. EGs are known to act preferentially on the amorphous water-binding parts of the cellulose fibres [35] and, therefore, their distinguished liquefaction potential can be explained by the loss of water-binding capacity and eventual collapse of the substrate upon EG action. By contrast, the CBHs, xylanases and βG had no substantial effect on the fluidity of the material. These results are consistent with our previous observations using purified enzymes from the industrially relevant fungus *T. reesei *in similar viscosity trials [22]. No clear difference between the EGII/Cel5A and EGV/Cel45A could be observed at 50°C or 60°C. The relative molar dosage of EGV/Cel45A was about 30% higher than that of EGII/Cel5A, thus reflecting a slightly better performance of the latter when used at the same protein dosage. Based on its temperature profile showing a clear decrease in viscosity even at 75°C, Ta EGII/Cel5A was chosen as the enzyme with the highest potential for efficient liquefaction at high temperatures. Primarily, the high thermostability of this EG type of enzyme can make a liquefaction stage possible at a notably elevated temperature. There is no direct comparison available in the literature on the hydrolytic performance of the two enzymes, but At EGV/Cel45A has recently been compared with EGII enzymes from other thermophilic sources [36]. At EGV/Cel45A was found to solubilize at least twice as much sugar from phosphoric acid-swollen cellulose, Avicel or bacterial cellulose as the *Myceliphtora thermophila *EGII/Cel5A after 24 hours of hydrolysis, but was approximately half as efficient as the *Thielavia terrestris *EGII/Cel5A in similar hydrolysis studies based on protein content.

The efficient hydrolytic performance of the enzyme(s) used in the pre-hydrolysis step is important. In an ideal case, the liquefying component(s) potentially already present in the pre-hydrolysis mixture would contribute to a significant extent to the efficient saccharification of the substrate, and thus reduce the overall enzyme requirement. Owing to the inherent synergistic behaviour of the various types of hydrolytic enzymes [37-39], it may be beneficial to use a dedicated mixture during the pre-hydrolysis step, thus allowing maximized exploitation of complementing hydrolytic activities. With this in mind, the combined hydrolytic performance of thermostable enzymes was studied in mixtures containing an enhanced proportion of the key liquefying enzyme, Ta EGII/Cel5A, in various ratios with the other components. Based on the protein content, the mixtures contained the same amount of EG as used in the RVA studies, to ensure at least as efficient liquefaction as obtained previously. The EG:CBH ratio was kept constant, and the mixtures were supplemented with the XYL and βG preparations.

The saccharification properties of mixtures containing the different CBH enzymes differed significantly, and the order of their performance also varied depending on the temperature. The results were not fully consistent with the optimum temperatures of the CBH enzymes determined by Voutilainen *et al. *[14] using the initial hydrolysis rate of single enzymes at various temperatures. In that study, using a short time activity assay (10 minutes) against the soluble substrate methylumbelliferyl lactoside (MUL), the Ct CBHI was always superior to the other thermostable CBH enzymes at temperatures from 30°C to 75°C. However, we found At CBHI/Cel7A to be clearly the best-performing CBH on a solid substrate up to a temperature of 55°C, above which the activity decreased.

The addition of xylanase improved the saccharification performance of mixtures in all combinations, with better performance achieved using Ta XYL/Xyn10A. The mechanism most probably involved an increase in free cellulose surface by removal of the residual hemicellulose from the pretreated fibres, thus helping the action of the cellulases synergistically, as suggested by the results of GC analysis.

The mixtures used in this study contained an enhanced ratio of the key liquefying enzyme, EGII/Cel5A, to achieve efficient reduction of substrate viscosity in the high-solid process. The mixtures were not optimized for efficient saccharification, thus the relatively low CBH content and the lack of CBHII and other minor activities (accessory enzymes) were expected to limit the hydrolytic performance considerably. Nevertheless, some of the more efficient mixtures outperformed the commercial reference enzymes at higher reaction temperatures.

## Conclusions

In this study, heterologously expressed enzymes from various thermophilic organisms were compared in the liquefaction of hydrothermally pretreated wheat straw using a real-time viscometric technique. Of the studied enzymes, EGs gave the most pronounced reduction in viscosity of the substrate, even during the first hour of the hydrolysis. EGII/Cel5A from *T. aurantiacus *was efficient up to 75°C. The performance of various thermostable CBHI/Cel7A enzymes was compared in mixtures containing a high proportion of the key liquefying enzyme. The most efficient CBH enzyme was At CBHI/Cel7A. The optimal combination of primary liquefying and secondary saccharifying enzymes can be expected to result in a reduced total enzyme use and correspondingly decreased costs in the production of bioethanol from high-solid lignocelluloses.

## Competing interests

The authors declare that they have no competing interests.

## Authors' contributions

NS planned and coordinated the experiments, carried out viscosity measurements and hydrolysis studies, analyzed the results, and drafted the manuscript. EH performed viscosity trials and hydrolysis experiments. JZ helped in the carbohydrate analysis. TP helped in the selection and characterization of thermostable enzymes. MSa and LV conceived and coordinated the overall study, and helped to analyze the results and finalize the paper. All authors critically revised the draft and approved the final manuscript.
